# Re-Licious: Co-Design with Adolescents to Turn Leftovers into Delicious and Healthy Meals—A School-Based Pilot Intervention

**DOI:** 10.3390/ijerph20166544

**Published:** 2023-08-08

**Authors:** Eva L. Jenkins, Linda Brennan, Michaela Jackson, Tracy A. McCaffrey

**Affiliations:** 1 Department of Nutrition, Dietetics and Food, Monash University, 264 Ferntree Gully Road, Melbourne 3168, Australia; 2 School of Media and Communication, RMIT University, Melbourne 3000, Australia; linda.brennan@rmit.edu.au (L.B.); michaela.jackson@rmit.edu.au (M.J.)

**Keywords:** co-design, behaviour change, food waste, healthy eating, adolescents, school

## Abstract

One-third of the food produced globally is lost or wasted, and one cause is consumer leftovers. Re-licious was an eight-week pilot intervention aiming to increase awareness of food waste and healthy eating by building adolescents’ ability to prepare and cook leftovers. Re-licious used a co-design approach and was piloted in a secondary school, half of which was during a COVID-19 lockdown period. Students watched videos on food waste and healthy eating during class. They identified leftover ingredients at home and repurposed ingredients to create recipes. Students co-created recipe criteria to ensure the personal relevance of the recipes. They completed pre- and post-intervention questionnaires (*n* = 40) about food waste and motivation and interest in healthy eating. Four group interviews were conducted. The factors identified as important in the co-creation sessions were preparation time, cost, healthiness, and sustainability. Participants with low motivation and interest in healthy eating decreased, and participants with high interest increased (*p* < 0.001). The intention to reduce food waste increased (*p* = 0.007), as did resourcefulness (*p* < 0.001) and personal norms (*p* = 0.048). Interviews highlighted the students’ increased awareness of food waste and enjoyment of the intervention. With improvements based on this pilot, Re-licious could be adapted and re-trialled in a face-to-face format to educate young people about food waste.

## 1. Introduction

Food waste reduction is becoming an increasingly urgent public health and environmental issue across the globe. Food waste occurs when edible food is thrown away in the retail or consumption stage of the food supply chain [1]. The Food and Agriculture Organization of the United Nations estimates that one-third of food produced globally is lost or wasted [1]. Sending food to landfill produces potent greenhouse gases, such as methane and carbon dioxide, and constitutes 8–10% of global emissions [2]. Furthermore, food waste is estimated to cost the global economy one trillion US dollars annually [2] and can cost individuals up to 35% of their food budget [3]. The United Nations has recognised the detrimental effects of food waste on both the economy and the environment [4]. In response, Sustainable Development Goal 12 aims to halve global food loss and waste by 2030 [4]. Working with young people in settings they naturally spend time in, such as school, is key to achieving this target [5]. Awareness of food waste from an early age could ensure that young people form lifelong sustainable habits, thereby creating meaningful long-term change in the food waste landscape and promoting a future sustainable food system [6].

The causes of food waste range from individual to wider societal factors. A major proportion of food waste research has focused on the individual level, assessing the determinants of food waste behaviour rather than focusing on solutions to food waste from a broader view [7]. Furthermore, most food waste interventions have traditionally focused on providing the household’s dietary ‘gatekeeper’ (i.e., the person who organises food and meals in the house) with information about how to reduce food waste [7]. However, other population groups, such as adolescents, are also key to the issue [8]. Adolescents have the power to influence their family’s attitudes, beliefs, and sustainable behaviours [9,10], potentially contributing to larger-scale changes, such as the changes in the overall household’s food-related knowledge [7], awareness [7], or food waste [8]. 

Limited research, particularly in Australia, has focused on exploring schools as an element of the behavioural ecology for food waste education [7,11,12]. Prior research has also shown that teaching students about health and nutrition at school can create positive changes in their home environment [13]. At school, both teachers and peer groups influence adolescents at a time when they are conscious of social norms and are beginning to develop lifelong health behaviours [14]. Thus, schools are ideal for delivering healthy lifestyle and sustainability-focused interventions outside the family and in an environment in which young people naturally spend time [15]. Moreover, students can transfer their knowledge to their parents and community, creating wider positive changes in their social environment [10,16]. 

Previous research has demonstrated that schools are a promising setting to educate young people on the environment [10] and food waste [12]. In Australia, specific objectives relate to teaching sustainability in the school curriculum; however, the scope is broad and individual topics, such as food waste, are not mentioned in the curriculum guidelines [17]. The school setting more broadly is key to supporting students to acquire knowledge and practically apply their knowledge [18]. Therefore, school is a key setting that should be utilised for food waste education. However, providing students with information alone is insufficient to tackle food waste [11]. Students must have an interest in food waste and want to learn the necessary skills at school to reduce food waste in their home and maintain their behaviours over time [11]. 

Many health promotion initiatives value individual participation as a vital component of developing skills and successfully changing behaviour [19]. In a school-based setting, student participation involves the students having agency (i.e., influence over decisions and activities in the school’s processes) rather than just taking part [19]. Co-design and participatory design methods provide equal value to expertise derived from lived experience and that from education and recognise that people are experts in their own lives [20]. These methods involve people creating their own solutions to problems, ensuring that the outcome is relevant to their needs [20], and have been shown to increase students’ agency by increasing engagement, satisfaction, motivation, skills, competency, and knowledge [19,21]. Co-design has been successfully used in a school setting through conducting multiple workshops to explore adolescents’ food choices, as well as physical and mental health needs [22]. Specifically, the workshops aimed to gather potential solutions that could be implemented in a future health literacy intervention to ensure it was relevant to the target population [22]. Successful researcher-led food waste interventions have changed knowledge, attitudes, or behaviour across a range of target audiences including: food service workers [23]; teacher and students [24,25,26]; and parents and students [11,12]. However, there is further potential to engage students in the co-design process to create their own intervention outcomes and ensure the solutions created for food waste reduction are relevant to them. 

Given that reducing leftovers is considered one of the most effective strategies to reduce household food waste [27,28], it is surprising that, of the six studies mentioned above, only one [11] prioritised discussing leftovers with students and their parents. Leftover consumption varies between individuals because of the many reasons that leftovers are wasted. For example, there is stigma attached to leftovers with many people seeing them as undesirable because they are ‘second-hand’ [29] and preferring to eat new, fresh food [30]. Others believe that leftovers are lower quality [31], boring, and do not taste nice [27], or they simply waste leftovers due to concern over food safety [32]. The challenges of leftover consumption need to be addressed to achieve significant consumer food waste reduction. Therefore, a novel pilot intervention, Re-licious, was developed to combine multiple areas that are relatively underexplored in the context of food waste: school-based research, co-design methods, and leftover education and consumption. 

### Purpose

This paper aims to describe the methodology, findings, and key learnings from Re-licious, a school-based pilot intervention. Specifically, the objectives of the pilot intervention were to:

1. Increase adolescents’ awareness and knowledge of food waste and healthy recipe creation:
2. Increase adolescents’ ability to use leftovers:
3. Reduce adolescents’ self-reported food waste;
4. Gather insights and feedback from participants regarding their involvement in the intervention. An outcome of the intervention was the creation of an ebook using the recipes created by the adolescents.

## 2. Materials and Methods

### 2.1. Study Design

**Figure 1:**
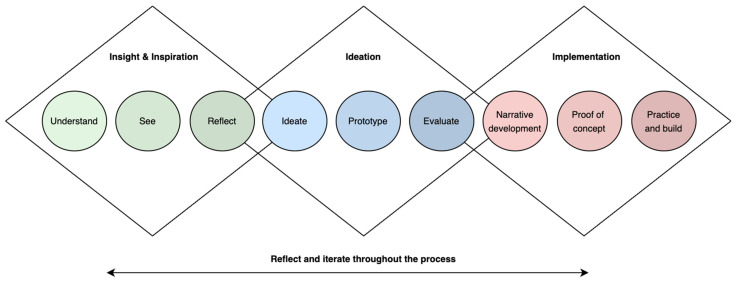
A co-design methodology, adapted from Lombard et al. [33] and IDEO [34].

Re-licious was a pilot intervention that used quantitative and qualitative methods and was run as a case study at one independent school in Melbourne, Victoria, Australia. Ethical approval was granted by the Monash University Human Research Ethics Committee (ID: 29748). Consent forms were sent to parents and students prior to the project. The intervention followed a co-design process and comprised three main phases: insight and inspiration, ideation (i.e., the formation of ideas or concepts), and implementation (Figure 1) [33,34]. Reporting followed the guidelines for group-based behaviour change interventions from the EQUATOR Network [35].

### 2.2. Participants

**Figure 2:**
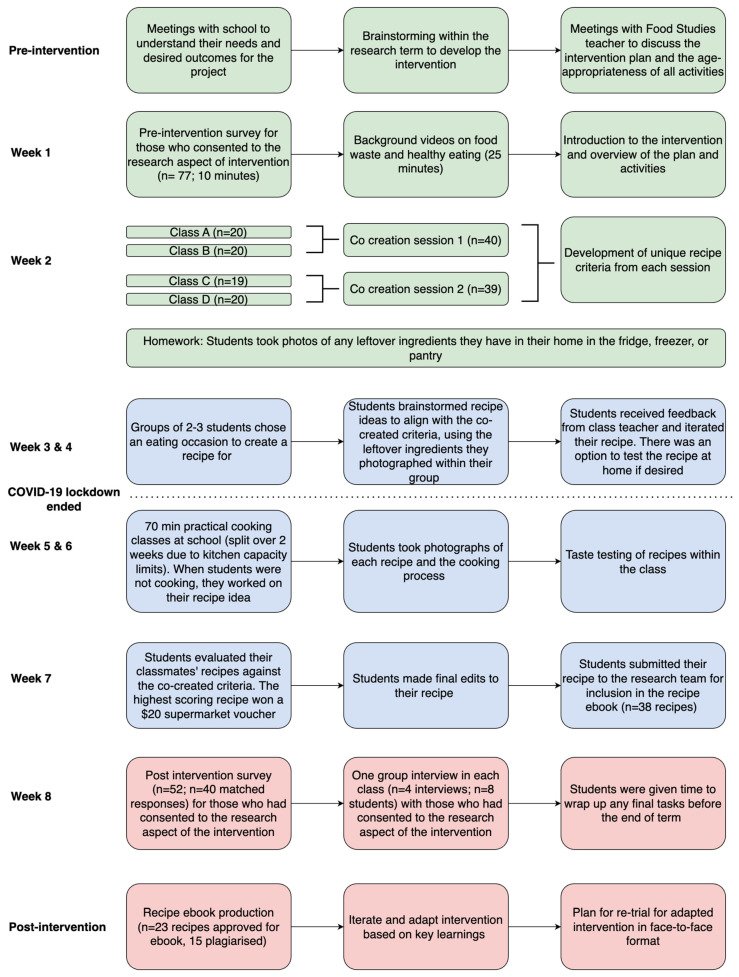
Overview of the key steps in the Re-licious co-design intervention across the eight-week period. The coloured boxes refer to the co-design phase: green = insight and inspiration; blue = ideation; red = implementation.

The inclusion criteria were Year 8 Food Studies students from the participating school (13–14 years old; *n* = 79). The exclusion criteria included those who did not assent or have parental consent to participate in the research component of the study. Those who did not provide consent undertook the same classes but did not complete the research aspect (i.e., survey and interviews). Figure 2 details the number of participants in each component of the intervention.

### 2.3. Procedure 

Data collection occurred during Term 4 (October to December) 2021, which included eight weeks of classes (one class of approximately 70 min per week). The Food Studies teacher (female, tertiary-educated) facilitated all classes and met the investigator (E.L.J.) weekly to discuss the upcoming intervention activities. The intervention was completed during class as part of the standard curriculum for the Food Studies unit. As a result of COVID-19 restrictions, the beginning of the intervention was delivered through online learning, and the second half was delivered in person. Melbourne schools have pivoted to online classes before; so, the teacher was well-equipped to deliver the intervention content in this way. 

The students completed a survey in the first week of the intervention (Figure 2). The survey was designed using Qualtrics (Provo, UT, USA) survey software. Prior to administering the survey to students, E.L.J. met with the Food Studies teacher to discuss its suitability for adolescents, who confirmed that it was age-appropriate. Survey topics included demographics, interest and motivation in health and healthy eating assessed by the Living and Eating for Health Segments (LEHS; see Supplementary File S1 for descriptions of each segment) [36], cooking and food preparation skills [37], food waste knowledge and attitudes [38], and self-reported food waste based on the five most commonly wasted categories of food, categorised by their AUD value [39,40] (Supplementary File S2). Students then watched pre-recorded videos developed by E.L.J. (approximately 25 min) about food waste and healthy eating based on the Australian Guide to Healthy Eating [41]. Next, the students formed groups of two or three and decided whether to create a recipe for breakfast, lunch, dinner, dessert, or snack. Students were instructed to take photographs at home of any leftover ingredients in their refrigerator, pantry, or freezer. 

The following week, E.L.J. and the Food Studies teacher ran two online co-creation sessions with the students. There were different students in each of the two sessions with attendance based on the Food Studies class they were in. Both sessions followed the same format. The co-creation activity aimed to develop ‘good’ recipe criteria that would guide the students’ recipe creations throughout the term. Each student was asked, ‘What are some things that are important to you when making a recipe?’ and provided five answers via the Mentimeter word cloud software. The Food Studies teacher and E.L.J. asked follow-up questions about common answers to form a clear recipe criteria that students could follow. For example, if the students listed ‘time’ as important, E.L.J. asked the group how long would be appropriate for recipe preparation and cooking. An anonymous poll was created with the top 10 options suggested by the students to reach a consensus on the final recipe criteria. 

During the following week, the students shared photographs of their leftover ingredients with their group. Their challenge was to use only the photographed ingredients to create an original recipe that would meet the criteria defined in the co-creation sessions. Students were granted two weeks to research and ideate their final recipe for the eating occasion they chose (breakfast, lunch, dinner, snack, or dessert). They were then provided 70 min in the school kitchen to prepare the recipe they designed and to take photographs of the creation process.

The students submitted their final recipes to the research team, who created an ebook containing the recipes. The students completed a post-survey questionnaire, and E.L.J. conducted four semi-structured interviews with student pairs (*n* = 8). The interviews took approximately 15 min and aimed to understand the students’ experiences as participants (see Supplementary File S3 for the interview guide).

### 2.4. Data Analysis 

Quantitative data were analysed using SPSS (Version 28.0., New York, NY, USA). Participants who did not complete the survey at both time points (*n* = 37) were excluded from the analysis, leaving 40 complete responses. Negatively worded variables within the scales were reverse coded. Cronbach’s alpha was calculated to assess the reliability for all scales, with a value above 0.5 considered an acceptable reliability level because each scale had fewer than 10 items [42]. For scales deemed reliable (*n* = 7, see Supplementary File S2), a mean score for the overall scale was calculated by summing the individual items and dividing them by the number of items in the scale. A paired sample *t*-test was used to compare the mean scale score between time point 1 (pre-intervention) and time point 2 (post-intervention). For ordinal variables, the related-samples Wilcoxon signed-rank test was used to assess significant differences. Significance was set at *p* < 0.05. 

Qualitative data were transcribed using Otter.ai technology, and interview transcripts were analysed using NVivo qualitative analysis software (Release 1.6.2). Interviews were coded and analysed by E.L.J. using inductive thematic analysis, following the guidelines developed by Braun and Clarke [43]. Whilst E.L.J. conducted the analysis, codes were discussed frequently with the co-authors during the coding process. Respondent validation could not be conducted as the research team did not have direct access to students’ contact details and interviews were conducted at the end of a school-term, after which students would be in the next year level. As there were a small number of interviews (*n* = 4) and they were shorter than expected (approximately 15 min due to time constraints), there were insufficient data to support an in-depth thematic framework. Therefore, the results are presented based on the interview guide questions that elicited rich participant insights.

## 3. Results

The Re-licious intervention was delivered across four classes in Year 8 Food Studies with a total of 40 students consenting to the research aspect of the intervention and completing both surveys. More participants identified as male (52.5%) than female (45.0%) and gender fluid (2.5%), and all were aged either 13 years (40%) or 14 years old (60%). A total of 38 recipes were submitted, with 15 identified as being from other published sources and not included in the final recipe ebook. Further details of the recipes are not included in this paper.

The results are presented in three sections: co-created recipe criteria, survey findings, and participant feedback. 

### 3.1. Co-Created Recipe Criteria 

The two co-creation sessions aimed to engage students in the intervention and encourage them to participate in forming the direction of the intervention. The co-creation sessions were based around defining a clear recipe criteria for students to follow throughout the program.

The criteria created by the first session ensured that the recipes included different skill levels, dietary requirements, and resources (Table 1). In contrast, the second session highlighted that low-cost, sustainable, and healthy recipes were important. They recognised that eating sustainably is essential to reduce their impact on the planet, and they discussed their consideration of which animal products they choose to consume. Feedback from the students indicated that they found the criteria helped them to understand the intervention and kept them on track throughout the recipe creation process. 

Co-creation demonstrated that each group of students was motivated by different factors when creating a recipe. The implementation of ideas and concepts that the students co-created into the intervention plan (e.g., using the recipe criteria as a design brief) ensured that subsequent activities were relevant and meaningful to them and hence increased their engagement with the intervention.

### 3.2. Survey Findings 

Across the intervention, there was a significant increase in personal norms related to throwing food away (i.e., participants were more conscious of throwing food away; *p* = 0.048; Table 2). Furthermore, participants’ intention to reduce food waste significantly increased across the program (*p* = 0.007; Table 2). There was also a mean increase in resourcefulness, which was related to participants being more confident in preparing a healthy meal with limited ingredients as well as using leftovers to create a new meal (*p* < 0.001; Table 2). On average, self-reported skills and beliefs related to behavioural control over food waste, consumer awareness of food waste, and cooking methods increased, but the differences were not significant (Table 2).

The most notable change in self-reported food waste behaviour was the number of participants wasting ‘quite a lot of food’, which reduced from two to zero (Table 3). Similarly, there was a slight increase in the number of participants wasting no food at all; however, the difference was not significant (*p* = 0.653). Further details on self-reported food waste separated by food category can be found in Supplementary File S4. 

Understanding how people view the importance of health-related behaviours in their lives can be informative when developing effective strategies to promote public health. Using the LEHS, most participants at the start of the intervention identified as ‘Balanced All Rounders’ (45%), followed by ‘Health Conscious’ (20%; Table 4). The distribution changed when assessed at the end of the intervention, with 40% of participants identifying as ‘Health Conscious’ and 17.5% identifying as ‘Balanced All Rounders’ (*p* < 0.001). A key difference was 15% of participants moving from ‘Contemplating Another Day’ to other, more engaged, LEHS (*p* < 0.001).

Overall, the quantitative findings demonstrate that Re-licious was successful in achieving Objectives 1 and 2 due to improving adolescents resourcefulness, personal norms, and intention to avoid food waste. However, Objective 3 of reducing self-reported food waste was not achieved. 

### 3.3. Participant Feedback 

As previously mentioned, the interviews were shorter than expected due to class time restraints. Therefore, to explore Objective 4, the results are presented in the following sections based on four key topics in the interview guide that engaged the adolescents (Supplementary File S3): highlights and challenges of the intervention, changed perspectives on leftovers, and the use of the recipe ebook.

#### 3.3.1. Highlights of the Intervention 

The students enjoyed learning about food waste and felt excited to participate in classes that differed from their usual curriculum. The content covered in the Re-licious intervention was personally relevant and engaging to the students because they co-created the recipe criteria, which informed their recipes throughout the program. Overall, the students had positive attitudes towards the activities in the Re-licious program (Quote 1, Table 5). 

The combination of learning and applying knowledge related to sustainability and food waste within the Re-licious intervention was new to the students and provided them with the knowledge and confidence to reduce their food waste in the future (Quote 2, Table 5).

The students also enjoyed recipe ideation and creation within the co-design process. Self-efficacy was further increased by practically applying preparation and cooking skills throughout the intervention. The students reflected on their intention to use leftover ingredients at home with their newfound resourcefulness (Quote 3, Table 5).

The enjoyment that came from learning about food waste, practising cooking skills, and having the ability to cook at home from leftover ingredients was a key highlight of Re-licious from the students’ perspective and demonstrates that the intervention achieved Objectives 1 and 2. 

#### 3.3.2. Challenges of the Intervention 

The main challenge during the Re-licious intervention was the need for online learning due to the COVID-19 lockdown. Feedback indicated that the intervention would have been more engaging if it was in person for the entire duration. The students found it harder to communicate with their peers during the lockdown period, but adapted to the situation and ensured that they met online to continue their group work (Quote 4, Table 5). The students were also concerned that they would not be able to create their recipes in the school kitchen due to the lockdown (Quote 5, Table 5). 

Despite the uncertainty, the kitchen classes went ahead in person as the COVID-19 lockdown ended. The COVID-19 lockdown was also challenging from the research team’s perspective, as the participating school had strict requirements that visitors were not allowed on school grounds for the duration of the year 2021. This meant the intervention plan had to be adapted and the research team could not attend as many classes as originally planned, for example, the kitchen practical classes. Despite the challenges identified, the Food Studies teacher was highly experienced and managed the challenges to ensure the intervention was delivered according to plan. 

#### 3.3.3. Changed Perspectives on Leftovers and Food Waste from the Intervention

The students were asked how Re-licious changed the way they saw leftovers. Many students were unaware of the negative consequences of food waste prior to the intervention. They referenced an increased awareness and understanding of leftovers and food waste at an individual and a broader scale (Quote 6, Table 5).

The students further reflected on their intention to use leftovers; they felt empowered and confident to reduce or completely combat food waste after learning about its detrimental effects (Quote 7, Table 5). 

Similarly, the students who were interviewed discussed their changing attitudes and beliefs towards leftovers across the school term as they applied their knowledge practically throughout the recipe creation process (Quote 8, Table 5). 

Many students considered their future food waste behaviour and started to make changes. They provided examples of specific situations where they will use their learnings, such as when they are at home on the weekend looking for something to eat (Quote 9, Table 5).

Overall, the students indicated that Re-licious changed their perceptions of leftovers and potentially their use of leftovers in the future.

#### 3.3.4. Use of the Co-Designed Recipe Ebook 

After the Re-licious program finished, a recipe ebook was created and provided to students so they could continue practising their cooking skills at home. The students were asked how they would use the ebook in the future. Feedback indicated they were interested in recreating ideas from their peers to see how they taste and felt excited about seeing the recipes in the ebook (Quote 10, Table 5). 

Many students indicated that the ebook would be useful at various times, such as weekends and school holidays. The ebook offered them confidence to cook on their own, knowing that they have access to a range of simple and age-appropriate recipes that use common leftover ingredients in the house (Quotes 11 and 12, Table 5).

The participants discussed both the highlights and challenges of the intervention (Objective 4). The feedback highlights that, from the students’ perspective, Re-licious was a successful and engaging pilot intervention. The students’ feedback suggested improvements to the pilot that could be made in future adaptations, such as conducting the intervention in a face-to-face format.

## 4. Discussion

### 4.1. Summary of Findings 

To our knowledge, Re-licious was the first pilot intervention of its kind to work with students in the school setting to co-design healthy, delicious recipes from leftover ingredients. Re-licious supported students to use their knowledge in an engaging, hands-on way to ensure that, by the end of the intervention, they had the skills required to use their learnings from the school setting in their home environment. The students reported a significant increase in their intention to avoid food waste, their resourcefulness, and personal norms relating to food waste, achieving Objectives 1 and 2. There was a significant change in students’ interest and motivation for health and healthy eating; many selected a LEHS [36] that reflected an increased interest in health post-intervention. Whilst there was no significant change in self-reported food waste (Objective 3), the participants indicated that they would rethink their typical eating behaviours (e.g., making instant noodles after school) in favour of a more sustainable choice (e.g., eating leftovers already in the house). Participant feedback highlighted the challenges of the intervention related to COVID-19 and provided ideas for future adaptations (Objective 4). 

Re-licious increased students’ knowledge of food waste and positively influenced their attitudes towards leftovers. The use of leftovers and the idea of reducing food waste was likely normalised due to ongoing class discussions during the eight-week pilot intervention. Other school-based interventions have also successfully changed students’ knowledge and attitudes towards food waste; however, they did not use a co-design methodology [12,24,25]. One food waste reduction program in primary schools successfully changed knowledge and attitudes towards food waste: students viewed food waste as detrimental to the environment post-intervention [24]. It is important to work with students in primary and secondary schools to enhance their knowledge of food and sustainability [16]. While research is still emerging on school-based food waste interventions, these interventions can positively affect the knowledge, attitudes and personal norms of participating students. However, as was found by others, converting food waste knowledge to food waste reduction remains a challenge, particularly in the medium and long term [25]. Future interventions should consider a co-design approach to ensure the content is relevant, engaging, and empowering so that students have more control over their food-related habits and can contribute to a sustainable future.

The students’ resourcefulness and self-efficacy significantly increased across the intervention, likely because of the focus on using leftovers to create and prepare a recipe. Qualitative feedback indicated that students created different meals to make from leftovers post-intervention, which is important for creating independence and healthier food habits. Past longitudinal research has reported that adolescents who help to prepare dinner multiple times a week are more likely to enjoy cooking, write shopping lists, and prepare dinner with meat or vegetables in emerging adulthood (i.e., 19–23 years) [44]. Despite the aforementioned behaviours, there were limited associations between adolescent cooking involvement and overall diet quality in adulthood (i.e., older than 23 years) [44]. In contrast, the cross-sectional research by Lavelle et al. found that both child (younger than 12 years) and teen (13–18 years) learners have greater cooking and food skills, diet quality, and overall health than people who learn these skills as adults [45]. Food preparation and cooking skills are required to prepare nutritious meals, and people that learn these skills earlier are more likely to create lasting healthy habits. Re-licious could be considered in a younger audience than adolescents to ensure that young people have the ability to prepare fresh food, reduce food waste, and live a healthy life.

The students in the Re-licious pilot intervention highlighted their desire to create healthy and sustainable recipes. The recent literature surrounding climate change has discussed the need to transition away from processed and animal-based products and move towards consuming healthy plant-based whole foods [46]. Traditionally, adolescents have poor dietary habits, such as the high consumption of sugar-sweetened beverages and a tendency to skip breakfast [47]. Despite this, the co-created recipe criteria in Re-licious showed that many students would prefer to include fresh, healthy, locally sourced ingredients in their recipes when they have the option. Many students also selected a segment post-intervention that reflected higher interest and motivation in health and healthy eating. This was also reflected in the qualitative discussions, where students discussed that they would be more likely to cook with their leftovers post-intervention than turn to other options, such as instant noodles. While the LEHS has been previously validated in terms of construct and nomological validity for assessing motivations and interest in health and healthy eating [36], this is the first time it has been used to assess changes in an intervention. 

Multiple reviews of school-based interventions have found that, among programs intended to change health attitudes and behaviours, multifaceted interventions were most frequently successful [48,49]. So, too, were those that incorporated teachers or staff members to facilitate the program [48,49]. In particular, home economics teachers were key facilitators of three studies conducted between 2005 and 2010 [50,51,52], two of which improved adolescents’ dietary intake [51,52]. Home economics teachers also facilitated a program that did not affect behaviour but improved awareness of the nutritional guideline ‘five a day’ [50]. Re-licious did not measure diet quality but rather interest and motivation around healthy eating using the LEHS, which could be a precursor to future healthy eating behaviours. Adolescents have an important role to play in facilitating a widespread shift to sustainable diets [53]; so, it is encouraging that they value both their health and creating healthy and sustainable recipes. Future iterations of Re-licious could measure dietary intake to understand whether changes in interest and motivation in health and healthy eating could translate to improvements in adolescents’ dietary intake.

Participants’ engagement with food waste was enhanced by the intervention. The students mentioned that they enjoyed the change from the regular curriculum and felt empowered to reduce food waste after Re-licious finished. Prior research gathered from a qualitative sample has also found that students are excited and engaged when learning about sustainability and enjoy having agency over their behaviour [26]. As mentioned, the co-creation activity was engaging for students participating in Re-licious. When students participate in class and are engaged with the content, they are more likely to perform better in their assessments, which creates a positive feedback loop and results in students being more engaged because it helps their grades [54]. By creating engaging classes that are co-created with the students, it is more likely that students will learn and remember the content. They could then change their behaviour outside of class to reflect their learnings, potentially contributing to the overall household food waste reduction. Future school-based interventions should prioritise design thinking and participatory co-creation activities to increase students’ engagement with the curriculum. 

### 4.2. Limitations

There were limitations throughout the Re-licious pilot intervention. The COVID-19 lockdown and subsequent restrictions meant that visitors were not allowed to attend the school campus. Hence, researchers could not attend classes to work directly with the students, affecting the ability of the research team to be fully immersed in the intervention and build rapport with participants. Instead, the researchers communicated regularly with the Food Studies teacher to ensure the content was delivered according to plan. 

While self-reported food waste was estimated by the participants, it was not directly measured because of time and budget restraints. On reflection, the questions used to assess food waste may not have been sensitive (i.e., too broad) or could have been confusing for the participants (e.g., estimating food waste in cups by food category). Additionally, the drop-out rate was high for the pre- and post-intervention survey (48%) which raises concern over the validity of the results. Conducting research with adolescents is challenging and the drop-out rate came down to students not being bothered to complete the follow-up survey. Therefore, statistical differences and changes in food waste volumes should be interpreted with caution. Furthermore, because of the limited number of interviews that could be completed in the timeframe, the qualitative feedback presented is indicative of some of the experiences of participating students and do not necessarily represent all participants.

### 4.3. Key Learnings from the Re-Licious Pilot Intervention 

The findings of this pilot intervention provide evidence that food waste could become a valued part of the school curriculum to promote health and sustainability. However, the cost of running a school-based intervention is a major factor when considering wider implementation. Teachers are typically time-poor and, therefore, the intervention must provide enough value to the participants and the participating school in order to be considered viable. From the limitations identified, we developed five key learnings for future iterations of the Re-licious intervention. While these learnings are specific to Re-licious, they may assist other researchers in planning a school-based intervention.

1. Measure food waste directly if resources allow for it. Re-licious relied on self-reported food waste, which can be challenging for adolescents to estimate, particularly if they are not in control of the food in their household. In future, it would be beneficial to involve the participants’ parents to gather their estimates on household food waste. If possible, conducting visual audits or physically measuring food waste in the household would be ideal to measure any changes in household food waste across the intervention period. This would minimise self-reporting bias, which is common in surveys.
2. The time taken to obtain parental consent and student assent must be considered before running a school-based intervention. For Re-licious, consent forms were emailed to parents weeks before the school term began, and it took multiple reminders before parents completed the forms. Some parents did not complete the form, which meant that the students could not participate in the research aspect of the intervention. In future, it would be best to distribute the consent forms in the previous term (i.e., 2–3 months before the intervention commences) to ensure there is adequate time to contact all parents. Another key consideration is whether the intervention will run in a government school or an independent/Catholic school because there are different permissions and processes to facilitate research.
3. The most engaging aspect of the intervention based on weekly meetings with the Food Studies teacher was the practical cooking classes. The teacher commented that it would be beneficial to have more than one practical class to break up the research phase of the intervention because one term of Food Studies typically includes at least two practical sessions. The second practical class could be conducted in the first few weeks of term and focus on a zero-waste recipe challenge (e.g., students must use all parts of the meat/vegetables) or have a health focus (e.g., substitute ingredients in a recipe to make it healthier).
4. Each student group created different meals with different ingredients, which made the food ordering process difficult for the school. Because the school orders ingredients for practical classes from a wholesale supplier with minimum quantities of each ingredient required, Re-licious could inadvertently lead to more food waste. Our solution was to encourage students to bring any dry leftover ingredients from home (e.g., flour and dry rice), and the school provided fresh fruit, vegetables, and meat so that food safety was not compromised. Students were encouraged to bring containers to take cooked food home (provided it did not need refrigeration) and share it with friends and family.
5. Whilst the recipe ebook was not the main focus of this manuscript, of the 38 final recipes submitted to the researchers, 15 were copied from other sources and were therefore omitted from the published recipe ebook. We believe this resulted from a misunderstanding of the brief, with students thinking they could search online for, and submit, a recipe that contained their exact leftover ingredients. In future studies, it is imperative that plagiarism is discussed, and if students use a published recipe, they must cite the source, adapt the recipe, and not publish the recipe externally or claim the recipe as their own.

## 5. Conclusions

The school setting enables public health practitioners to engage with young people and provide education on issues such as health and sustainability. The Re-licious pilot intervention successfully changed students’ attitudes towards food waste by increasing their resourcefulness, intention to reduce food waste, and motivation and interest in health and healthy eating. Students enjoyed the change in curriculum and were interested in learning about food waste. The qualitative feedback indicated that learnings at school were transferred to the home environment, with some participants explaining how they would use their new skills to use up leftover ingredients. The co-creation of the recipe criteria was effective in ensuring that the content was engaging and that the recipes created were relevant to students’ needs. Future school-based interventions could implement a co-design methodology using both quantitative and qualitative methods to obtain important insights into the success of the intervention. Overall, Re-licious holds promise in increasing students’ resourcefulness and self-efficacy in using leftovers to create recipes and reduce food waste. With improvements based on the key learnings from this pilot, Re-licious could be adapted and re-trialled in a face-to-face format to educate more young people about healthy eating and food waste. 

## Figures and Tables

**Table 1:** Recipe criteria from the two co-creation sessions. The sessions had different students in them based on the Food Studies class that the student was in.

Criteria	Session 1 (*n* = 40)	Session 2 (*n* = 39)
Provide **recipe name, ingredient list, number of servings, methodology, and photographs**.	**😀**	**😀**
**Time.** Time taken for the recipe should be between 30–45 min to allow for cooking and cleaning during class.Snacks and breakfast should be quicker to make than lunch, dinner, and dessert.	**😀**	**😀**
**Difficulty level.** It should be clear whether the recipe is easy, moderate, or hard. The students decided upon the difficulty of the recipe as it is a subjective measure.	**😀**	
**Accessibility of ingredients and appliances.** Ingredients should be available from the local supermarket, and appliances should be common or provide alternatives (e.g., option to use a whisk instead of a hand mixer).	**😀**	
**Allergies/dietary requirements.** List the allergens and provide substitutions where possible to cater for dietary restrictions.	**😀**	
**Cost.** The recipe should cost under AUD 5 per serve (USD 3.40).		**😀**
**Healthy.** The recipe should be healthy, defined as the students having a balance of at least three food groups, including fruit or vegetables.		**😀**
**Sustainable.** The recipe should be sustainable, defined as having appropriate serving sizes so that no waste is left, having minimal animal products or considering the type of meat (i.e., red meat vs chicken), and, if possible, using local and in-season ingredients.		**😀**

**😀** signifies whether the co-created criteria were applicable to Session 1 and/or Session 2.

**Table 2:** Mean scores (SD) for food waste, cooking skills, and food skills scales during the pre- and post-8-week Re-licious intervention (*n* = 40).

	Pre-Intervention	Post-Intervention	*p* Value ^b^
**Food waste** ^a^			
Intention to avoid food waste	3.84 (0.71)	4.13 (0.76)	0.007
Perceived behavioural control	2.82 (0.65)	2.99 (1.09)	0.266
Personal norms	3.78 (0.84)	3.99 (0.90)	0.048
**Cooking skills** ^a^			
Cooking methods	3.70 (0.86)	4.03 (0.84)	0.072
Food preparation methods	3.49 (0.87)	3.78 (0.86)	0.077
**Food skills** ^a^			
Resourcefulness	3.20 (1.01)	3.77 (0.96)	<0.001
Consumer awareness	3.91 (0.91)	3.95 (1.22)	0.855

Results reported as mean (SD) from a 5-point scale, where 1 is ‘strongly disagree’ and 5 is ‘strongly agree’, for attitude-based questions, and where 1 is ‘very poor’ and 5 is ‘very good’ based on their confidence level for skill-based questions. ^a^ Full descriptions of the items in each scale are listed in Supplementary File S2. ^b^ The paired samples *t*-test was used to test for significant differences.

**Table 3:** Self-reported food waste before and after the 8-week Re-licious intervention (*n* = 40).

Food Waste Amount	Pre-Intervention n (%)	Post-Intervention *n* (%)	*p* Value ^a^
None	3 (7.5%)	4 (10.0%)	0.653
A small amount	20 (50.0%)	20 (50.0%)	
Some	12 (30.0%)	10 (25.0%)	
A reasonable amount	3 (7.5%)	6 (15.0%)	
Quite a lot	2 (5.0%)	0	

^a^ The related-samples Wilcoxon signed-rank test was used to test for significant differences.

**Table 4:** Interest and motivation in health and healthy eating from the Living and Eating for Health Segments [36] before and after the 8-week Re-licious intervention (*n* = 40).

Living and Eating for Health Segment ^a^	Pre-Intervention *n* (%)	Post-Intervention *n* (%)	*p* Value ^b^
Lifestyle Mavens	3 (7.5%)	7 (17.5%)	<0.001
Health Conscious	8 (20.0%)	16 (40.0%)	
Aspirational Healthy Eaters	5 (12.5%)	9 (22.5%)	
Balanced All Rounders	18 (45.0%)	7 (17.5%)	
Contemplating Another Day	6 (15.0%)	0	
Blissfully Unconcerned	0	1 (2.5%)	

^a^ Segments are listed from the highest to the lowest in terms of ability, interest, and motivation in health and healthy eating. See Supplementary File S1 for full segment descriptions. ^b^ The related-samples Wilcoxon signed-rank test was used to test for significant differences.

**Table 5:** Feedback from the participants.

Quote Number. Gender, Age	Participant Quote
**3.3.1 Highlights of the intervention**	
1. Female, 14 years	It was like something new that we’ve, like, never done. And we hadn’t done it in previous classes. That seemed cool. I really liked doing this because it was, like, a nice change from the regular lessons that we had.
2. Female, 14 years	I think it’s good that we’re learning about, like, we’re acknowledging the fact because that’s, like, the first steps to helping problems. And so doing it in school, you’re acknowledging and working on a task about it, and you learn more about these problems. And you just like, know, like problems, like cool, and like we will fix them, but at least we know that there’s a problem there to begin with.
3. Male, 14 years	I enjoyed, like, coming up with all the different recipes that we could have at our house, like just before, I wasn’t really sure of, like, what I could make with just a limited amount of ingredients at home. But now when I go home, I can see, ‘Oh I can make a pasta’, or something like that.
**3.3.2 Challenges of the intervention**	
4. Male, 13 years	[Online learning] was a little bit hard because you got distracted easily. But we would get on a Zoom call together to do our work so that we could talk to each other.
5. Female, 14 years	Pretty much the only challenge was being sure that we would be like at school to like, create the dishes, because it was still like lockdown.
**3.3.3 Changed perspectives on leftovers and food waste from the intervention**	
6. Female, 14 years	I think you’re just more aware of things that you could use that you don’t, and you’re just like, ‘Oh, I could have used this to do blah blah blah, or this or this’. And that would have been fun and easy, but I didn’t. And so, it’s kind of like, oh, you know, do it while you can. And it’s like a bigger problem when you realise … because it all adds up.
7. Female, 14 years	After this, it has, like, made me think about leftovers. Like, I could, like, do something with it instead of wasting it. And yes, just made me think of that being, like, as sustainable as possible.
8. Male, 14 years	There’s, like, much more to do with them [leftovers]. Like, say, you have some things in there that you won’t eat, you can actually use that to make something with it. And like, put it to good use, I guess, instead of leaving it there, which is really good.
9. Male, 13 years	If I’m at home on the weekend, instead of just grabbing a pack of two-minute noodles, I think and see, ‘Oh, I’ve got this leftover. I can make something with that’.
**3.3.4 Use of the co-designed recipe ebook**	
10. Male, 13 years	I think it’d be really cool to see other people’s ideas and make them, so you know how they taste and everything.
11. Female, 13 years	I feel like an ebook can be useful like for my family and myself when we go on holiday. So, like places where I don’t have much food, that we can use those recipes. Also when my parents are working, that I can make lunch for myself and stuff like that.
12. Female, 14 years	Personally, I have like a lot of free time on like, holidays, and weekends. So if I’m ever feeling bored, I could decide to make something for like me and my family.

## Data Availability

Data are available upon request from the corresponding author. The data are not publicly available due to ethical reasons.

## Supplementary Materials

The following supporting information can be downloaded at: https://www.mdpi.com/article/10.3390/ijerph20166544/s1. Supplementary File S1: The Living and Eating for Health Segments and descriptions; Supplementary File S2: Scales and items used in the pre- and post-intervention survey; Supplementary File S3: Semi-structured interview guide; Supplementary File S4: Self-reported food waste by food category.
