# Supplementary material for: Re-Licious: Co-Design with Adolescents to Turn Leftovers into Delicious and Healthy Meals—A School-Based Pilot Intervention
Source: Int J Environ Res Public Health. 2023 Aug 8;20(16):6544. doi: 10.3390/ijerph20166544 (PMC10454923; doi:10.3390/ijerph20166544)
Supplement: Supplementary file 1 [file ijerph-20-06544-s001.zip › Supplementary File S1.pdf]

Supplementary File S1. The Living and Eating for Health Segments and descriptions.

Table S1. Living and Eating for health segments and descriptions, to assess motivation and interest in health and healthy eating [36]. Participants chose one option and were exposed to the description only, not the segment name.

**Segment**	**Description**
Lifestyle Mavens	I'm passionate about healthy eating and health plays a big part in my life. I use social media to follow active lifestyle personalities or get new recipes/exercise ideas. I may even buy superfoods or follow a particular type of diet. I like to think I am super healthy.
Health Conscious	I'm health-conscious and being healthy and eating healthy is important to me. Although health means different things to different people, I make conscious lifestyle decisions about eating based on what I believe healthy means. I look for new recipes and healthy eating information on social media.
Aspirational Healthy Eaters	I aspire to be healthy (but struggle sometimes). Healthy eating is hard work! I've tried to improve my diet, but always find things that make it difficult to stick with the changes. Sometimes I notice recipe ideas or healthy eating hacks, and if it seems easy enough, I'll give it a go.
Balanced All Rounders	I try and live a balanced lifestyle, and I think that all foods are okay in moderation. I shouldn't have to feel guilty about eating a piece of cake now and again. I get all sorts of inspiration from social media like finding out about new restaurants, fun recipes and sometimes healthy eating tips.
Contemplating Another Day	I'm contemplating healthy eating but it's not a priority for me right now. I know the basics about what it means to be healthy, but it doesn't seem relevant to me right now. I have taken a few steps to be healthier but I am not motivated to make it a high priority because I have too many other things going on in my life.
Blissfully Unconcerned	I'm not bothered about healthy eating. I don't really see the point and I don't think about it. I don't really notice healthy eating tips or recipes and I don't care what I eat.
