# Supplementary material for: Re-Licious: Co-Design with Adolescents to Turn Leftovers into Delicious and Healthy Meals—A School-Based Pilot Intervention
Source: Int J Environ Res Public Health. 2023 Aug 8;20(16):6544. doi: 10.3390/ijerph20166544 (PMC10454923; doi:10.3390/ijerph20166544)
Supplement: Supplementary file 1 [file ijerph-20-06544-s001.zip › Supplementary File S2.pdf]

Supplementary File S2. Scales and items used in the pre- and post- intervention survey

Attitude and skill-based questions

Table S2. Scales and items used in the pre- and post-intervention survey to assess food waste attitudes, norms, and cooking skills. Participants responded to each item on a 5-point Likert scale ranging from strongly disagree (1) to strongly agree (5) for attitude-based questions, and a scale of very poor (1) to very good (5) based on their confidence level for skill-based questions.

Scale and items	Cronbach Alpha in this study
**Intention to avoid food waste (n=4 items)** [38]	
(1) I try to waste no food at all.	
(2) I always try to eat all purchased foods.	
(3) I try to produce only very little food waste.	
(4) I aim to use all leftovers.	0.880
**Perceived behavioural control (n=3 items, adapted to be relevant to adolescents by removing 2 items)** [38]	
(1) I find it difficult to prepare a new meal from leftovers[a].	
(2) I find it difficult make sure that only small amounts of food are discarded in my household[a].	
(3) I have the feeling that I cannot do anything about the food wasted in my household.	0.857
**Personal norms (n=4 items)** [38]	
(1) I feel bad when I throw food away.	
(2) I feel obliged not to waste any food.	
(3) It is contrary to my principles when I have to discard food.	
(4) I have been raised to believe that food should not be wasted and I still live according to this principle.	0.918
**Cooking skills**	
**Cooking Method (n=5 items, adapted to be relevant to adolescents by removing 3 items)** [37]	
(1) Chop, mix and stir foods, for example chopping vegetables, dicing an onion, cubing meat, mixing and stirring food together in a pot/bowl.	
(2) Blend foods to make them smooth, like soups or sauces' (using a whisk/blender/food processor etc).	
(3) Boil or simmer food (cooking it in a pan of hot, boiling/bubbling water).	
(4) Roast food in the oven, for example raw meat/chicken, fish, vegetables etc.	
(5) Fry/stir-fry food in a frying pan/wok with oil or fat using the gas rings/hot plates.	0.756
**Food Preparation Techniques (n=5 items)** [37]	
(1) Bake goods such as cakes, buns, cupcakes, scones, bread etc., using basic/raw ingredients or mixes.	
(2) Peel and chop vegetables (including potatoes, carrots, onions, broccoli).	
(3) Prepare and cook raw meat/poultry/fish.	0.675

(4) Make sauces and gravy from scratch (no ready-made jars, pastes or granules).	
(5) Use herbs and spices to flavour dishes.	
**Food skills**	
Meal planning and preparing **(n=3 items)** [37]	0.100 (not used in subsequent analysis)
(1) Planning meals ahead? (e.g. for the day/week ahead).	
(2) Preparing meals in advance? (e.g. packing lunch).	
(3) Following recipes when cooking.	
Resourcefulness **(n=3 items, adapted to be relevant to adolescents by removing 1 item)** [37]	0.653
(1) Preparing or cooking a healthy meal with only few ingredients on hand.	
(2) Preparing or cooking a meal with limited time.	
(3) Using leftovers to create another meal.	
Label reading/consumer awareness **(n=3 items, adapted to be relevant to adolescents by removing 1 item)** [37]	0.767
(1) Reading the best-before date on food.	
(2) Reading the nutrition information on food labels.	
(3) Balancing meals based on nutrition advice on what is healthy.	

[a]Items were reverse coded in analysis. Visshers et al., measure was adapted to be relevant to adolescents by omitting personal attitudes, financial attitudes, perceived health risks, and subjective norms scales. Lavelle et al., measure was adapted to be relevant to adolescents by omitting shopping and budgeting scales.

Self-reported food waste questions

1. For the following, estimate the amount (in whole cups) of edible food that is thrown out in a typical week in your household. (e.g. Solid / liquid food left on dinner plates, food that is thrown into the compost, green bin, waste bin, tipped down the sink, fed to pets etc.). Enter one response for each food category.

	Less than one cup	1-2 cups	3-4 cups	5 cups	More than 5 cups
Bakery (e.g. Bread, muffins, cakes, pastries etc.)					
Dairy (e.g. Milk, cheese, eggs, butter, yoghurt etc.)					
Meat & seafood (e.g. Chicken, beef, mince, fish, sausages etc.)					
Packaged & processed (e.g. Long life food, tinned food, processed meats, frozen vegetables, biscuits, flour, coffee, cereal, potato chips, uncooked rice/pasta etc.)					
Fruit & vegetables (e.g. apples, potatoes, salad items, herbs etc.)					

Food categories are based on the five most wasted food categories from prior research by the Fight Food Waste Cooperative Research Centre [39].

2. Thinking about the different types of food wasted in the previous question, overall, how much uneaten food would you say you generally end up throwing away? Select one option.

None	A small amount	Some	A reasonable amount	Quite a lot

Question adapted from the WRAP UK Household Food & Drink 2012 survey [40].
