# Supplementary material for: Re-Licious: Co-Design with Adolescents to Turn Leftovers into Delicious and Healthy Meals—A School-Based Pilot Intervention
Source: Int J Environ Res Public Health. 2023 Aug 8;20(16):6544. doi: 10.3390/ijerph20166544 (PMC10454923; doi:10.3390/ijerph20166544)
Supplement: Supplementary file 1 [file ijerph-20-06544-s001.zip › Supplementary File S3.pdf]

Supplementary File S3. Semi-structured interview guide with questions and (logic)

Co-creation activity

1. How did you feel about the activity?
2. How did you feel about the final criteria that your class created?

Recipe planning

3. Tell me a bit about the process of recipe development using leftover ingredients (*lived experience).*
    a. What were the main challenges throughout the development?
    b. What were the highlights of the process?
4. How did you search for cooking information to do your research and develop your recipe?

The recipe and project overall

5. How do you feel about the final recipe that you created?
6. How did you get your family/or the people you live with involved or talk to them about the project? *(social norms/family communication)*
7. How did you share your project or recipe with other people outside your family? Were there any key platforms you used to communicate with each other or share what you were doing? *(possible additional intervention or leverage point)*
8. In general, how could we engage people your age over social media to be interested or learn more about food waste and recipe creation? *(possible additional intervention/leverage point)*
9. How will the recipe ebook of the classes creations be useful for you?

Overall

10. How has the program changed the way you see leftovers in your house? *(knowledge)*
11. Are there any other comments or feedback you would like to make about the intervention or that we haven't discussed yet?
