# Supplementary material for: Re-Licious: Co-Design with Adolescents to Turn Leftovers into Delicious and Healthy Meals—A School-Based Pilot Intervention
Source: Int J Environ Res Public Health. 2023 Aug 8;20(16):6544. doi: 10.3390/ijerph20166544 (PMC10454923; doi:10.3390/ijerph20166544)
Supplement: Supplementary file 1 [file ijerph-20-06544-s001.zip › Supplementary File S4.pdf]

Supplementary File S4. Self-reported food waste findings

Table S3. Self-reported food waste by food group category, pre- and post- the 8-week Re-licious intervention (n=40)[a]

	Bakery n(%)		Dairy & eggs n(%)		Meat & seafood n(%)		Packaged & processed n(%)		Fruit & vegetables n(%)	
	Pre-	Post-	Pre-	Post-	Pre-	Post-	Pre-	Post-	Pre-	Post-
**<1 cup**	19 (47.5)	20 (50.0)	22 (55.0)	21 (52.5)	19 (47.5)	19 (47.5)	19 (47.5)	21 (52.5)	13 (32.5)	17 (42.5)
**1-2 cups**	16 (40.0)	17 (42.5)	11 (27.5)	12 (30.0)	11 (27.5)	12 (30.0)	16 (40.0)	14 (35.0)	16 (40.0)	12 (30.0)
**3-4 cups**	3 (7.5)	2 (5.0)	7 (17.5)	6 (15.0)	7 (17.5)	6 (15.0)	4 (10.0)	4 (10.0)	9 (22.5)	8 (20.0)
**5 cups**	2 (5.0)	1 (2.5)	0	1 (2.5)	3 (7.5)	2 (5.0)	0	0	2 (5.0)	2 (5.0)
**>5 cups**	0	0	0	0	0	1 (2.5)	1 (2.5)	1 (2.5)	0	1 (2.5)

[a]significance testing was unable to be performed as the categorical variables included in this study are repeated measures and not dichotomous. Pre- refers to before the 8-week Re-licious intervention, and post- refers to after the 8-week Re-licious intervention.
